# Stage-Dependent Peri-Implant Bone Remodelling Under Occlusal Contact in a Murine Model

**DOI:** 10.1016/j.identj.2026.109795

**Published:** 2026-08-05

**Authors:** Ruolan Cao, Yoko Takemura, Yasuko Moriyama, Wenxuan Lai, Xi Chen, Ami Izumi, Mari Abe, Yasunori Ayukawa

**Affiliations:** Section of Implant and Rehabilitative Dentistry, Division of Oral Rehabilitation, Faculty of Dental Science, Kyushu University, Fukuoka, Japan

**Keywords:** Bone remodelling, Dental implant, Occlusal contact, Osseointegration, Peri-implant bone

## Abstract

**Introduction and aims:**

Occlusal contact conditions are dynamic during implant healing and maintenance, but peri-implant bone responses may depend on the maturity of the bone–implant interface. This study evaluated stage-dependent peri-implant bone responses to contact-level and loading-change conditions using a rat intraoral implant model.

**Methods:**

Fifty five-week-old male Wistar rats were allocated to 5 groups: 0.5, 1.0 and 1.5 groups, representing transmucosal segment heights of 0.5, 1.0 and 1.5 mm, respectively, and groups with ipsilateral or contralateral loading-change conditions after initial osseointegration (IL and CL, respectively). The 0.5, 1.0 and 1.5 groups were compared as healing-phase loading groups, and the 1.0, IL and CL groups as post-osseointegration loading-change groups. Outcomes included the bone area fraction (BA%), apparent bone–implant contact (BIC%), tartrate-resistant acid phosphatase (TRAP)- and alkaline phosphatase (ALP)-positive areas, and immunofluorescence for connexin 43 (Cx43) and sclerostin.

**Results:**

During healing, the 1.5 group showed higher BA% and ALP-positive area than the 0.5 group, but lower BIC% and more pronounced discontinuity at the crestal bone–implant interface. Cx43-positive area was higher in the 1.0 and 1.5 groups than in the 0.5 group, whereas sclerostin-positive area was lowest in the 1.0 group. After osseointegration, the IL and CL groups showed lower BIC% and greater TRAP-positive area than the 1.0 group, without a significant reduction in BA% over 1 week. Sclerostin corrected intensity was higher in the IL and CL groups than in the 1.0 group.

**Conclusion:**

Within the limitations of this rat model, peri-implant bone responses to occlusal contact-related conditions were stage dependent. Greater or preserved peri-implant bone area did not necessarily indicate continuous bone–implant contact, supporting the concept of bone area–interface decoupling.

**Clinical relevance:**

Implant occlusion should be managed as a daily, dynamic condition, with contact control during healing and continued reassessment during maintenance, even when bone levels appear stable.

## Introduction

Dental implants are extensively used to replace missing teeth and restore masticatory function, with high long-term survival rates.^1^ However, peri-implant diseases, marginal bone changes and prosthetic complications remain important challenges for the long-term maintenance of dental implants.^1^^,^^2^

Occlusion is a dynamic functional system involving teeth, periodontal tissues, mandibular movement and neuromuscular activity.^3^ For dental implants, occlusion should not be regarded merely as a static contact scheme established at prosthesis delivery. Rather, it represents a continuous functional condition created by mastication, parafunction, prosthetic wear, dentition changes and alterations in occlusal support during maintenance.^3^, ^4^, ^5^, ^6^, ^7^, ^8^ Occlusal contacts, prosthetic wear, dentition changes and parafunctional habits may evolve after prosthesis delivery and therefore require ongoing monitoring.^6^, ^7^, ^8^ In this context, understanding how peri-implant bone responds to loading-related conditions at different stages of implant therapy is clinically relevant.

For implant-supported restorations, occlusal contact level, crown-height space, prosthetic height, contact distribution and lever arms are clinically observable variables that may influence load transmission and mechanical stability.^3^^,^^4^^,^^9^^,^^10^ Although occlusal overload has long been discussed as a contributor to peri-implant bone loss, current evidence does not support a simple cause-and-effect relationship in humans. Its biological effect appears to depend on the loading magnitude, direction, duration, inflammatory status and biofilm exposure.^11^, ^12^, ^13^, ^14^, ^15^, ^16^ Thus, occlusal loading should be viewed as a dynamic maintenance issue rather than a single adjustment at prosthesis delivery.

Unlike natural teeth, implants lack periodontal ligament cushioning and proprioceptive feedback. Therefore, abrupt or excessive changes in occlusal contact may be less effectively buffered and may be transmitted more directly to the bone–implant interface and surrounding bone.^8^^,^^17^ Bone adapts to local mechanical stimuli,^18^ and peri-implant bone adaptation may involve changes in the bone structure, bone–implant contact, remodelling activity and osteocyte-mediated mechanosensing. Osteocytes coordinate skeletal responses to mechanical stimuli, in part, through intercellular communication and the regulation of Wnt-related bone formation.^19^, ^20^, ^21^, ^22^ Therefore, connexin 43 (Cx43) and sclerostin were selected in this study as exploratory markers of osteocyte-related mechanosensitive signalling. Changes in these markers can be interpreted as supportive evidence of altered mechanosensitive signalling rather than proof of a complete causal pathway.

Previous studies indicate that the loading magnitude and timing influence peri-implant bone behavior.^23^, ^24^, ^25^, ^26^, ^27^, ^28^ During early healing, excessive or premature loading may impair osseointegration, potentially through interfacial micromotion beyond the biological tolerance threshold.^23^^,^^24^^,^^27^^,^^28^ Conversely, with sufficient primary stability and appropriate prosthetic conditions, early loading may still yield acceptable clinical outcomes.^5^^,^^24^ Together, these observations suggest that the effect of loading is stage dependent and context dependent, particularly with respect to the maturity of the bone–implant interface and the biological endpoint used to evaluate the response.

Peri-implant bone area and bone–implant contact may reflect different aspects of peri-implant tissue status, but whether they respond in parallel during interface formation and after osseointegration remains unclear. Few studies have compared peri-implant bone responses during healing with those after osseointegration within the same experimental framework. Therefore, this study used a rat intraoral implant model with 2 experimental settings: healing-phase contact-level conditions created by varying the implant transmucosal segment height, and post-osseointegration loading-change conditions created by dentition alteration after initial osseointegration. The study aimed to determine whether the peri-implant bone structure, bone–implant contact, remodelling activity and exploratory osteocyte-related mechanosensitive markers differed between these settings. We hypothesised that peri-implant bone would show stage-dependent response patterns, providing biological support for stage-specific occlusal monitoring during implant therapy.

## Materials and methods

### Animals and experimental design

Fifty male Wistar rats (5 weeks old, approximately 120 g body weight) were used in this study. The animals were housed under standardised conditions with free access to water and a standard laboratory diet. Only male rats were used to reduce biological variability in this exploratory model; therefore, sex-related differences in peri-implant bone adaptation were not assessed. The experimental protocol was approved by the Animal Experiment Committee of Kyushu University (approval number: A21-368-0; approved on 26 July 2021) and conducted in accordance with the ARRIVE 2.0 guidelines.^29^

Animals were individually numbered and randomly assigned by a computer-generated sequence to 5 experimental groups: 0.5, 1.0, 1.5, ipsilateral change in occlusal loading (IL), and contralateral change in occlusal loading (CL) groups (n = 10 per group). In each group, 5 animals were allocated to undecalcified ground section analysis and 5 to undecalcified frozen section analysis. The 0.5, 1.0 and 1.5 groups constituted the healing-phase loading groups, in which functional contact conditions during healing were modified by varying the implant transmucosal segment height and occlusal contact level. The IL and CL groups constituted the post-osseointegration loading-change groups. The IL and CL groups received 1.0-mm implants, followed by ipsilateral or contralateral dentition alteration 4 weeks after implantation, respectively. Accordingly, each analytical dataset included 5 animals per group. In this model, 4 weeks after implantation was considered to represent initial osseointegration.

The sample size was based on previous similar animal studies and confirmed using the resource equation method. For each analytical dataset, 5 animals per group were included. With 5 groups, the E-value was 20 (N − G = 25 − 5 = 20). For each of the 2 three-group comparisons, the E-value was 12 (15 − 3 = 12), which is within the recommended range for the resource equation method.^30^ bone–implant contact (BIC%) was considered the main histomorphometric outcome because it reflects continuity at the bone–implant interface. Bone area fraction (BA%), tartrate-resistant acid phosphatase (TRAP)-positive area, and alkaline phosphatase (ALP)-positive area were secondary outcomes, and Cx43 and sclerostin immunofluorescence were exploratory outcomes. No animal, implant or specimen was excluded from the analysis.

Group allocation was not blinded during surgery because the transmucosal height of the implant was visually apparent. Histomorphometric and immunofluorescence images were coded before quantitative analysis to minimise observer bias.

### Surgical procedures

**Fig. 1:**
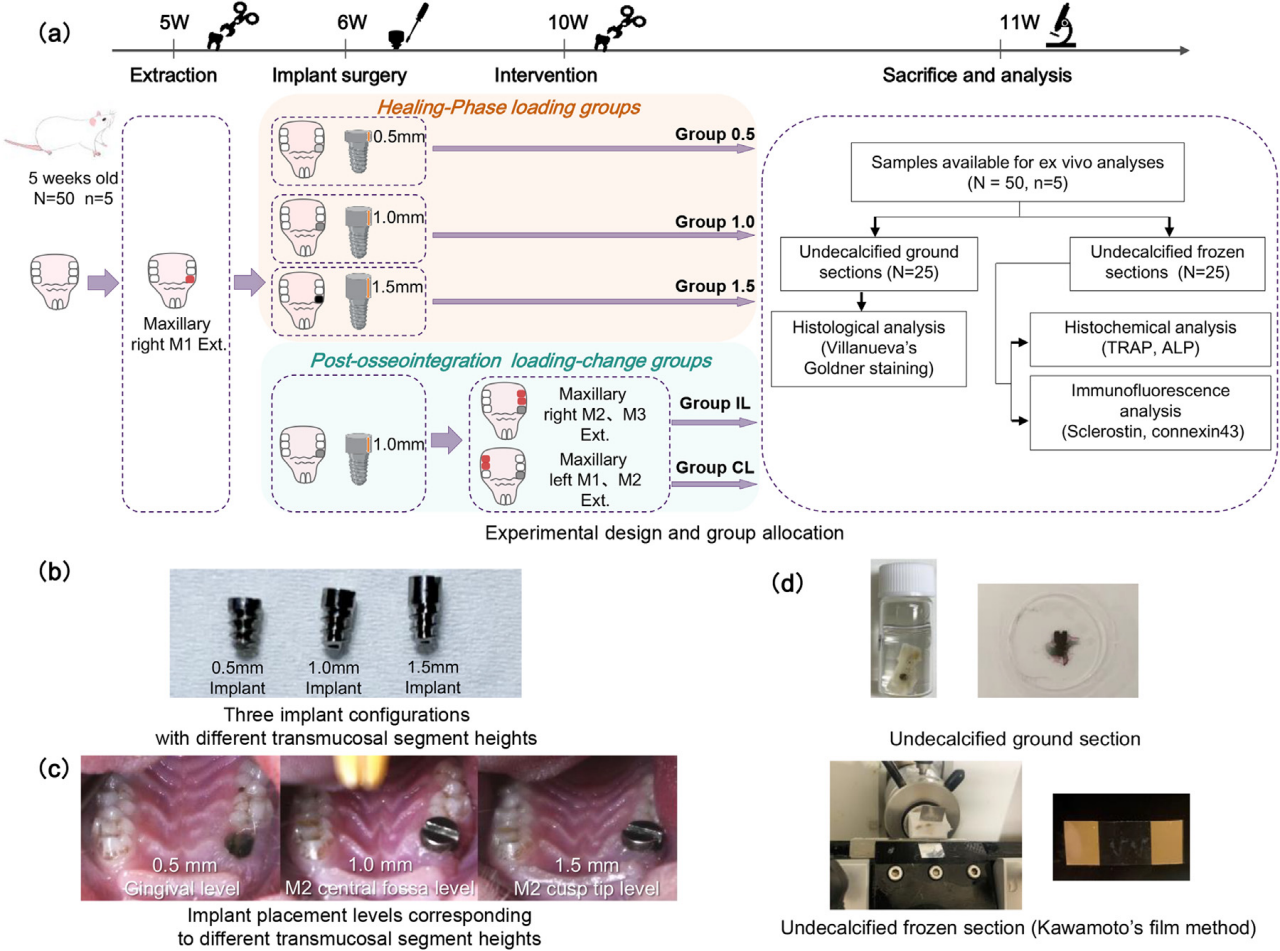
Experimental design**,** implant configuration**,** and specimen preparation workflow**.** (a) After extraction of the right maxillary first molar, implants with different transmucosal segment heights were placed to establish height-dependent occlusal contact-level conditions during the healing phase. 4 weeks after implantation, when initial osseointegration was considered to have been achieved, the intraoral occlusal environment was altered in the post-osseointegration loading-change model by extraction of the right maxillary second and third molars in the IL group or the left maxillary first and second molars in the CL group. Samples were harvested 5 weeks after implantation for histological, histomorphometric, TRAP/ALP, and immunofluorescence analyses. (b) Photographs and schematic drawings of the 3 implant configurations. The implants had different transmucosal segment heights but the same intraosseous length. (c) Representative immediate postoperative photographs showing implant placement levels. The highest points of the implants were aligned with the gingival level, the central fossa of the right maxillary second molar, or the cusp tip of the right maxillary second molar, corresponding to transmucosal segment heights of 0.5, 1.0, and 1.5 mm, respectively. (d) Representative specimens prepared for undecalcified ground-section analysis and Kawamoto’s film method-based undecalcified frozen-section analysis. Ext., extraction; IL, ipsilateral change in occlusal loading; CL, contralateral change in occlusal loading; ALP, alkaline phosphatase; TRAP, tartrate-resistant acid phosphatase.

All surgical procedures were performed under general anaesthesia using medetomidine (0.3 mg/kg), midazolam (4.0 mg/kg) and butorphanol (5.0 mg/kg). The right maxillary first molar was extracted atraumatically. One week later, implant beds were prepared at the extraction sites, and the implants were placed (Fig. 1a). Osteotomies were created by stepwise drilling with K-expanders (#80-#120; Maillefer, Ballaigues, Switzerland). Subsequently, screw-shaped commercially pure titanium implants (Sky Blue, Fukuoka, Japan; diameter, 2 mm; total length, 3.0, 3.5 or 4.0 mm; transmucosal segment, 0.5, 1.0 or 1.5 mm; intraosseous segment, 2.5 mm) with machined surfaces were inserted as previously described (Fig. 1b).^31^

Implants with different transmucosal segments heights were used to create different contact-level conditions. The implants were positioned according to the assigned group. In the 0.5 group, the highest point of the 0.5-mm implant was positioned at the gingival margin. In the 1.0, IL and CL groups, the highest point of the 1.0-mm implant was positioned at the level of the central fossa of the right maxillary second molar. In the 1.5 group, the highest point of the 1.5-mm implant was positioned at the cusp tip of the right maxillary second molar (Fig. 1c). These configurations were intended to modify the functional contact conditions. The actual occlusal force magnitude, direction, frequency and duration at the interface were not measured directly. Accordingly, although the 0.5, 1.0 and 1.5 groups are referred to as the healing-phase loading groups, they were described in terms of implant height and contact level rather than as quantified low-, moderate- or high-loading groups.

Four weeks after implantation, the right maxillary second and third molars were extracted from the IL group, and the left maxillary first and second molars were extracted from the CL group. Implant stability was assessed weekly by visual inspection and clinical mobility testing. Peri-implant soft tissues were examined for gross clinical signs of inflammation, including redness, swelling or exudation.

All animals were euthanised under anaesthesia 5 weeks after implantation. Specimens were fixed by transcardial perfusion via the left ventricle, first with heparinised saline, and subsequently with 4% paraformaldehyde (pH 7.4). The right maxillae were harvested en bloc for further analysis.

### Preparation of undecalcified ground sections and histomorphometric analysis

Specimens designated for undecalcified ground section analysis were fixed in 4% paraformaldehyde for 24 hours, dehydrated through a graded ethanol series, cleared in xylene, and embedded in methyl methacrylate (Fujifilm Wako Pure Chemical Corporation, Osaka, Japan). Following polymerisation, sections were prepared using a precision saw (Leica SP1600; Leica Microsystems GmbH, Wetzlar, Germany) and subsequently ground and polished (MetaServ 250; Buehler Ltd., Lake Bluff, IL, USA) to a final thickness of 50-70 µm. Sections were stained with Villanueva-Goldner stain, which renders mineralised bone green and soft tissues pink (Fig. 1d).

**Fig. 2:**
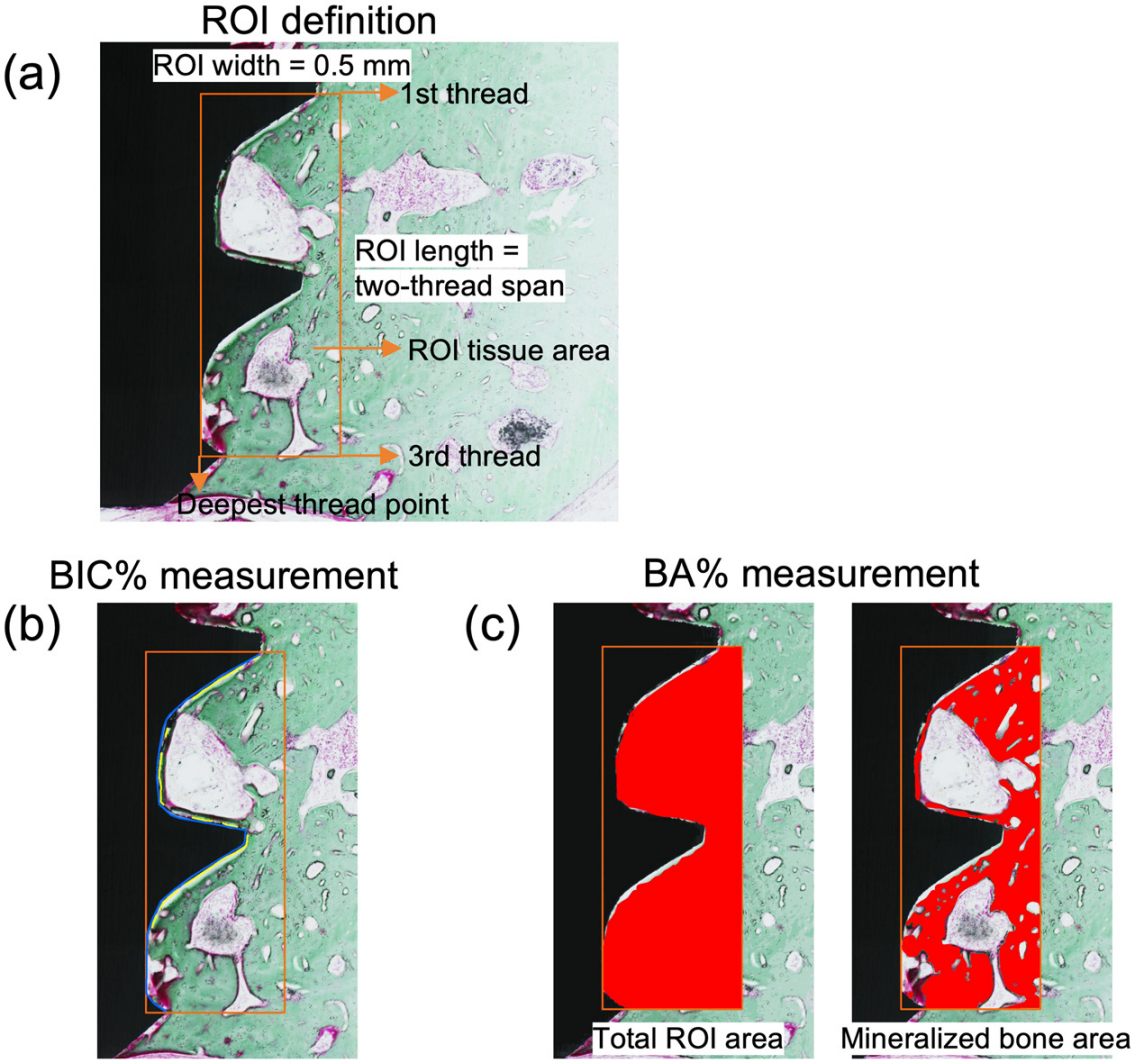
Definition of the region of interest and histomorphometric analysis. (a) The region of interest (ROI) comprised the tissue area enclosed by the rectangular boundary, extending from the first to the third implant thread and 0.5 mm laterally from the deepest point of the implant thread. (b) Measurement of bone–implant contact (BIC, %). The blue line indicates the total implant surface length, and the yellow line indicates the bone–implant contact length. (c) Measurement of bone area (BA, %). The total ROI area and the mineralised bone area within the ROI are illustrated.

The region of interest (ROI) extended longitudinally from the most coronal point of the first implant thread to that of the third thread and laterally 0.5 mm from the deepest point of the thread (Fig. 2). Histomorphometric parameters included the BA% and BIC%. BA% was defined as the mineralised bone area within the ROI divided by the total ROI area. Because no distinct boundary between pre-existing and newly formed bone was consistently identifiable, the BA% was interpreted as the bone area fraction within the ROI rather than as newly formed bone alone. BIC% was defined as the proportion of the implant surface that was in direct contact with the bone relative to the total implant surface length within the ROI. Histomorphometric terminology followed the American Society for Bone and Mineral Research guidelines.^32^

### Undecalcified frozen sections, histochemistry and immunofluorescence analysis

Undecalcified frozen sections were prepared using the Kawamoto film method^33^ to preserve the mineralised bone structure for histological and immunofluorescence analyses. Specimens were embedded in SCEM medium (Section-Lab Co., Ltd., Hiroshima, Japan), and Cryofilm Type 2C (9) (Section-Lab Co., Ltd.) was applied to the specimen surface during sectioning. Sections of 5 µm thickness were produced (Fig. 1e).

To assess osteoclastic and osteoblastic activity, sections were stained with a TRAP/ALP kit (Fujifilm Wako Pure Chemical Corporation) according to the manufacturer’s instructions. TRAP and ALP were used as histochemical markers of bone resorption and bone formation, respectively.^34^^,^^35^

For immunofluorescence analysis, sections were pretreated and blocked according to the manufacturer’s protocol and then incubated overnight at 4°C with primary antibodies against Cx43 (Proteintech Group, Rosemont, IL, USA; 26980-1-AP; 1:100) and sclerostin (Proteintech Group; 21933-1-AP; 1:100). Fluorescence detection was performed using the FlexAble 2.0 CoraLite system according to the manufacturer’s instructions, and nuclei were counterstained with DAPI. Negative controls were prepared by omitting the primary antibody while keeping all other steps, including FlexAble 2.0 CoraLite detection, washing, counterstaining and mounting, unchanged. These controls were used to assess tissue autofluorescence and nonspecific background fluorescence.

### Image acquisition and statistical analyses

Sections were examined under a light microscope, and images were analysed using Fiji software,^36^ an ImageJ distribution (National Institutes of Health, Bethesda, MD, USA). For undecalcified ground sections, 1 standardised field within the predefined peri-implant ROI was analysed per animal for the BA% and BIC%. For undecalcified frozen sections, 3 standardised, non-overlapping fields within the ROI were analysed per animal for TRAP, ALP, Cx43 and sclerostin, and averaged to obtain 1 animal-level value for each outcome. Thus, the animal was considered the experimental and statistical unit, with 5 animals per group used for each outcome.

All images within each staining batch were acquired using identical magnification, exposure time, gain and illumination settings. A single manual threshold, determined using the negative control and representative positively stained sections, was applied unchanged to all images within the same batch. The positive area was expressed as the percentage of threshold-positive pixels within the ROI. For Cx43 and sclerostin, the background-corrected mean fluorescence intensity was calculated as the corrected total fluorescence (CTF) normalised to ROI area. The CTF was calculated as the integrated density of the ROI − ROI area × mean background fluorescence.

Statistical analyses were performed using SPSS Statistics 31 (IBM Corp., Armonk, NY, USA). Data are presented as the mean ± standard deviation. Group comparisons were performed separately for the healing-phase loading groups and post-osseointegration loading-change groups. Normality was assessed using the Shapiro–Wilk test and Q-Q plots, and variance homogeneity was assessed using the Brown-Forsythe test. Depending on the data distribution and variance homogeneity, one-way ANOVA with Tukey’s post hoc test, Welch’s ANOVA with Games-Howell comparisons or the Kruskal–Wallis test with Dunn-Bonferroni comparisons were used. Because of the limited sample size, statistical findings were interpreted as exploratory and considered together with histological observations. *P* < .05 was considered statistically significant. Exploratory Spearman correlations between the BA% and BIC% were analysed separately for the 2 experimental comparisons.

## Results

### Clinical observations

**Fig. 3:**
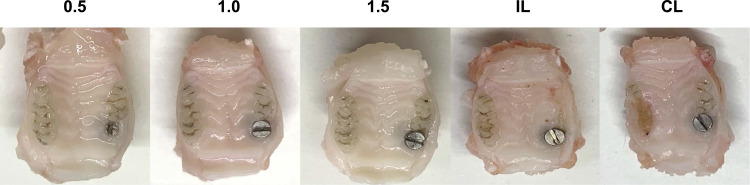
Representative clinical photographs of implant loading conditions and peri**-**implant soft tissue status in each experimental group. Representative clinical photographs show implant loading conditions and peri-implant soft tissue status at 5 weeks after implantation. In the IL and CL groups, the occlusal environment was altered 4 weeks after implantation, when initial osseointegration was considered to have been achieved, by extraction of the ipsilateral maxillary second and third molars or the contralateral maxillary first and second molars, respectively. No obvious peri-implant soft tissue inflammation or clinical mobility was observed in any group. IL, ipsilateral change in occlusal loading; CL, contralateral change in occlusal loading.

All 50 implants were successfully placed 1 week after tooth extraction. Then, 5 weeks post-implantation, the peri-implant soft tissues showed no gross clinical signs of inflammation, redness, swelling or exudation, and none of the implants displayed clinically detectable mobility (Fig. 3).

### Histological observations and histomorphometric analysis

Undecalcified ground sections revealed mineralised bone distributed along the implant surface in all specimens. Trabecular architecture was clearly discernible, with localised lamellar-like structures observed. There was no distinct boundary between pre-existing and newly formed bone, and no fibrous encapsulation was evident at the bone–implant interface.

**Fig. 4:**
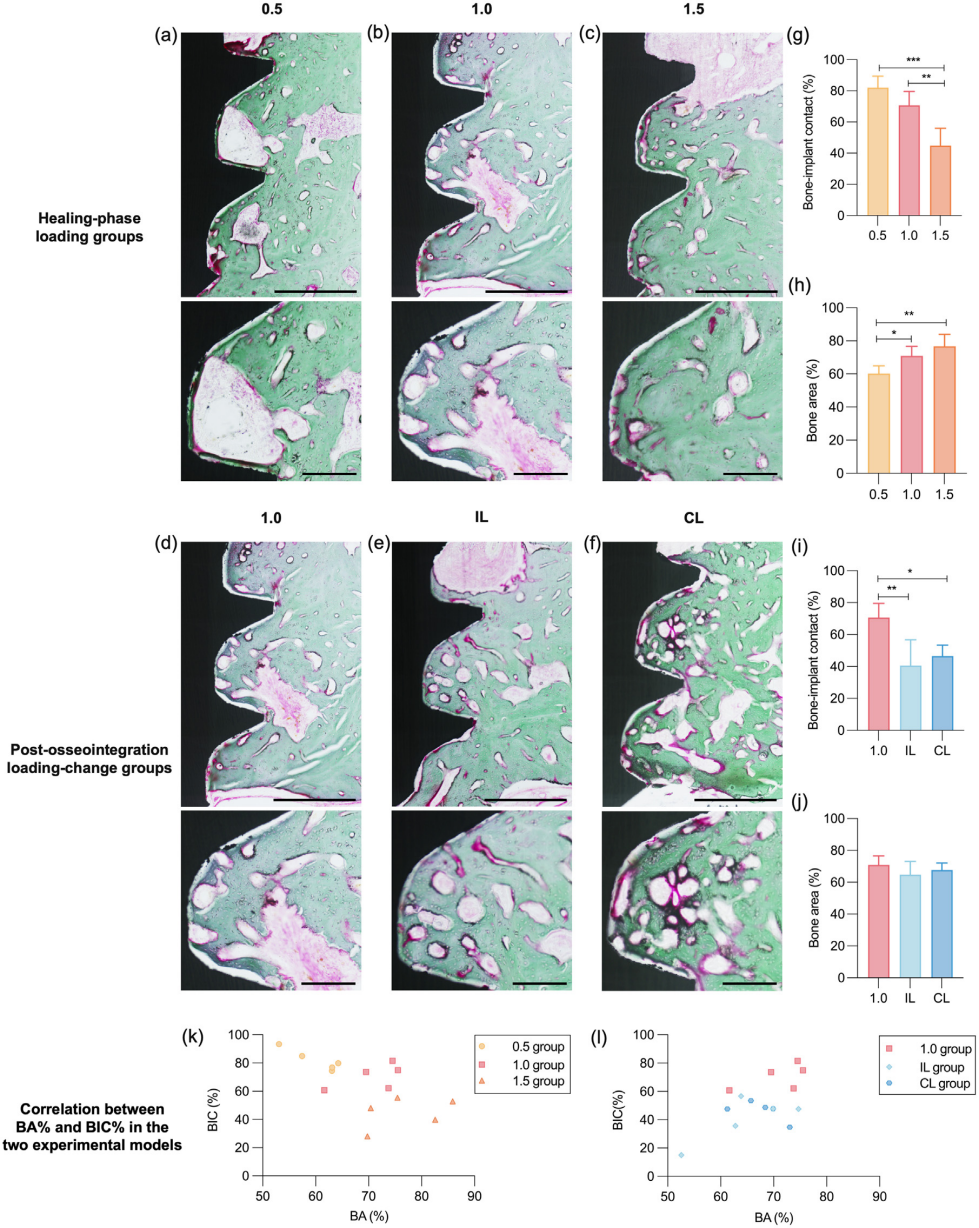
Histological and histomorphometric evaluation of peri**-**implant bone under different loading conditions. Representative Villanueva-Goldner-stained undecalcified ground sections are shown for the 0.5 group (a), 1.0 group (b), 1.5 group (c), 1.0 control group (d), IL group (e), and CL group (f). The 1.0 group served as the reference group for both the healing-phase loading model and the post-osseointegration loading-change model. Thus, panels b and d show the same representative image of the 1.0 group, with panel d presented as the 1.0 control group without dentition alteration for comparison with the IL and CL groups. Low- and high-magnification images are shown for each group. Scale bars, 500 μm and 200 μm, respectively. In the 0.5 group, marrow-like spaces were observed, whereas newly formed bone remained in close contact with the implant surface. The 1.0 and 1.5 groups showed denser newly formed bone and fewer marrow-like spaces. In the 1.5 group, soft tissue ingrowth was observed around the implant neck. In the IL and CL groups, small resorption cavities were observed around the bone–implant interface. Quantitative results are shown for BIC (%) in the healing-phase loading model (g), BA (%) in the healing-phase loading model (h), BIC (%) in the post-osseointegration loading-change model (i), and BA (%) in the post-osseointegration loading-change model (j). Spearman correlation analyses between BA (%) and BIC (%) are shown for the pooled healing-phase loading model (k) and the post-osseointegration loading-change model (l). Data are presented as mean ± SD. n = 5 animals per group for each analytical dataset. *P < .05, **P < .01, ***P < .001. IL, ipsilateral change in occlusal loading; CL, contralateral change in occlusal loading; BIC, bone–implant contact; BA, bone area.

In the healing-phase loading groups, the 1.5 group showed more pronounced reduced continuity of the crestal bone–implant interface than the 0.5 group. The resorption-like surfaces appeared irregular, with scattered lacunae. In some specimens, soft tissue ingrowth around the implant neck was observed, accompanied by localised discontinuities along the bone–implant interface. Peri-implant bone in the 1.5 group appeared denser, characterised by thickened trabeculae and reduced marrow-like spaces. Bone deposits along the implant surface were identified in localised areas (Fig. 4c). Conversely, the 0.5 group exhibited a comparatively loose bone structure, featuring thinner and more sparsely arranged trabeculae and larger marrow-like spaces, while maintaining a more continuous bone–implant interface (Fig. 4a).

Apparent BIC% differed significantly among the 0.5, 1.0 and 1.5 groups (0.5: 81.9 ± 7.5%; 1.0: 70.7 ± 8.9%; 1.5: 44.9 ± 11.1%; one-way ANOVA, *P* < .001). Post hoc analysis showed that the 1.5 group was significantly lower than the 0.5 and 1.0 groups (Tukey, *P* < .001 and *P* = .002), whereas the 0.5 and 1.0 groups did not differ significantly (*P* = .176) (Fig. 4g). BA% differed significantly among the 0.5, 1.0 and 1.5 groups (0.5: 60.2 ± 4.8%; 1.0: 71.0 ± 5.7%; 1.5: 76.8 ± 7.2%; one-way ANOVA, *P* = .003). Post hoc analysis showed higher BA% in the 1.0 and 1.5 groups than in the 0.5 group (Tukey, *P* = .036 and *P* = .002), with no significant difference between the 1.0 and 1.5 groups (*P* = .308) (Fig. 4h).

In the post-osseointegration loading-change groups, both the IL and CL groups showed more active remodelling-like changes at the bone–implant interface than the 1.0 control. Histological observations revealed localised bone resorption and decreased interface continuity. The surrounding cancellous bone exhibited irregular trabecular arrangement with focal disruptions. Localised bone cavities and small resorptive areas were observed around implants, although BA% was not statistically lower than in the 1.0 group (Figs. 4d, e).

Apparent BIC% differed significantly among the 1.0, IL and CL groups (1.0: 70.7 ± 8.9%; IL: 40.5 ± 16.1%; CL: 46.5 ± 7.0%; one-way ANOVA, *P* = .003). Post hoc analysis showed lower apparent BIC% in the IL and CL groups than in the 1.0 group (Tukey, *P* = .003 and *P* = .014), with no significant difference between IL and CL groups (*P* = .694) (Fig. 4i). In contrast, BA% did not differ significantly among the 1.0, IL and CL groups (1.0: 71.0 ± 5.7%; IL: 64.8 ± 8.3%; CL: 67.7 ± 4.5%; one-way ANOVA, *P* = .338) (Fig. 4j), indicating no statistically detectable reduction in peri-implant bone area over the 1-week observation period.

Exploratory Spearman correlation analysis was performed to examine the relationship between BA% and BIC%. In the healing-phase loading groups, BA% was significantly negatively correlated with BIC% in the pooled dataset (Spearman rho = -0.550, *P* = .034, n = 15; Fig. 4k), indicating that greater bone area fraction was not necessarily accompanied by greater apparent bone–implant contact. In the post-osseointegration loading-change groups, no significant correlation was observed between BA% and BIC% (Spearman rho = 0.438, *P* = .102, n = 15; Fig. 4l).

### TRAP and ALP staining

**Fig. 5:**
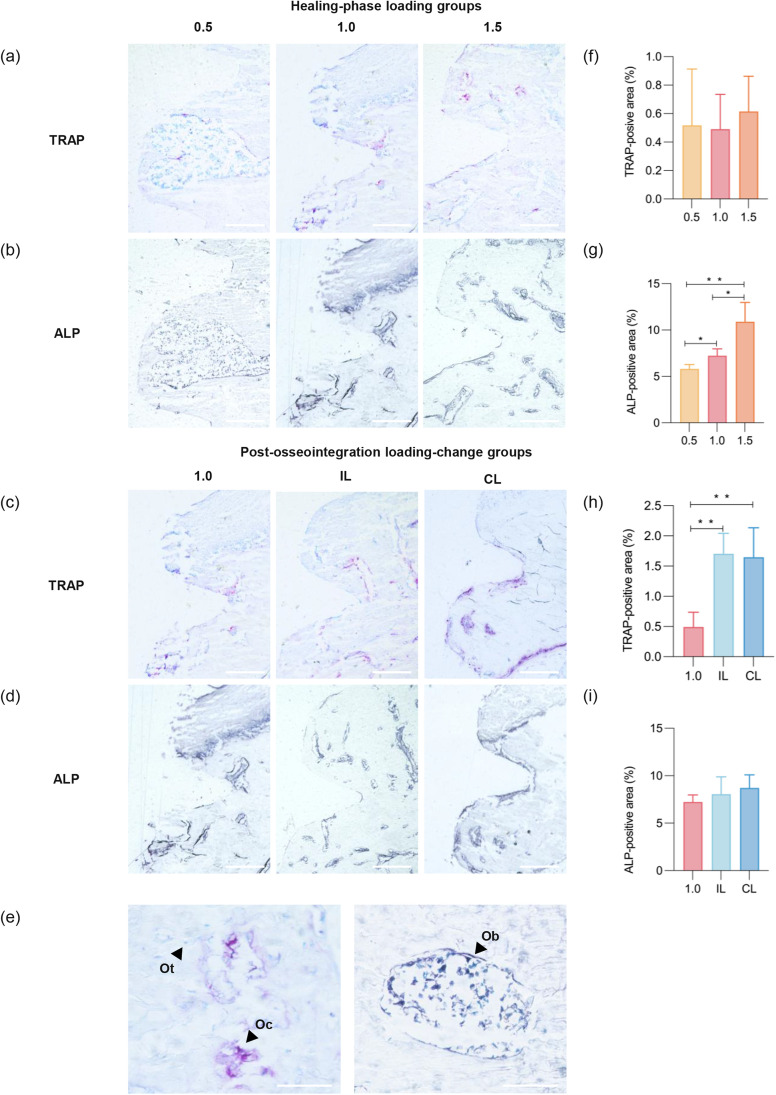
TRAP and ALP staining analyses. Representative TRAP- and ALP-stained images are shown for the healing-phase loading model and the post-osseointegration loading-change model (a-d). Because the 1.0 group served as the reference group for both models, the same representative TRAP- and ALP-stained images are shown for the 1.0 group and the 1.0 control group, with the latter presented for comparison with the IL and CL groups. Scale bars, 200 μm. High-magnification images of TRAP- and ALP-stained sections are shown in (e). Scale bars, 50 μm. Black arrows indicate representative osteocytes, osteoblasts, and osteoclasts, as labeled in the panels. Quantitative results are shown for TRAP-positive area (%) in the healing-phase loading model (f), ALP-positive area (%) in the healing-phase loading model (g), TRAP-positive area (%) in the post-osseointegration loading-change model (h), and ALP-positive area (%) in the post-osseointegration loading-change model (i). TRAP- and ALP-positive areas were quantified using Fiji and expressed as the percentage of positive staining area within the ROI. Data are presented as mean ± SD. n = 5 animals per group for each analytical dataset. *P < .05, **P < .01. TRAP, tartrate-resistant acid phosphatase; ALP, alkaline phosphatase; IL, ipsilateral change in occlusal loading; CL, contralateral change in occlusal loading; Ot, osteocytes; Oc, osteoclasts; Ob, osteoblasts.

In the healing-phase loading groups, TRAP-positive area was primarily localised along trabecular bone surfaces and within resorption lacunae (Fig. 5a). Quantitative analysis showed no significant difference in TRAP-positive area among the 0.5, 1.0 and 1.5 groups (0.5: 0.52 ± 0.39%; 1.0: 0.49 ± 0.24%; 1.5: 0.62 ± 0.25%; one-way ANOVA, *P* = .795; Fig. 5f). ALP staining appeared predominantly on bone surfaces and regions of active peri-implant bone formation, displaying either continuous or patchy patterns (Fig. 5b). The ALP-positive area differed significantly among the healing-phase loading groups (0.5: 5.81 ± 0.48%; 1.0: 7.23 ± 0.76%; 1.5: 10.91 ± 2.07%; Welch’s ANOVA, *P* = .002; Fig. 5g). Post hoc analysis showed that the 1.5 group was significantly higher than both the 0.5 and 1.0 groups, and the 1.0 group was also significantly higher than the 0.5 group (Games-Howell, *P* = .010, *P* = .030 and *P* = .024, respectively).

In the post-osseointegration loading-change groups, TRAP-positive area was primarily located at the bone–implant interface and along trabecular surfaces, occasionally forming clusters (Fig. 5c). Quantitative analysis showed that TRAP-positive area was significantly higher in both the IL and CL groups than in the 1.0 group (1.0: 0.49 ± 0.24%; IL: 1.70 ± 0.34%; CL: 1.65 ± 0.49%; Tukey’s test, both *P* < .001; Fig. 5h), whereas no significant difference was observed between the IL and CL groups (*P* = .970). In contrast, ALP-positive area did not differ significantly among the 1.0, IL and CL groups (1.0: 7.23 ± 0.76%; IL: 8.05 ± 1.83%; CL: 8.71 ± 1.38%; one-way ANOVA, *P* = .278; Fig. 5i).

### Immunofluorescence analysis

**Fig. 6:**
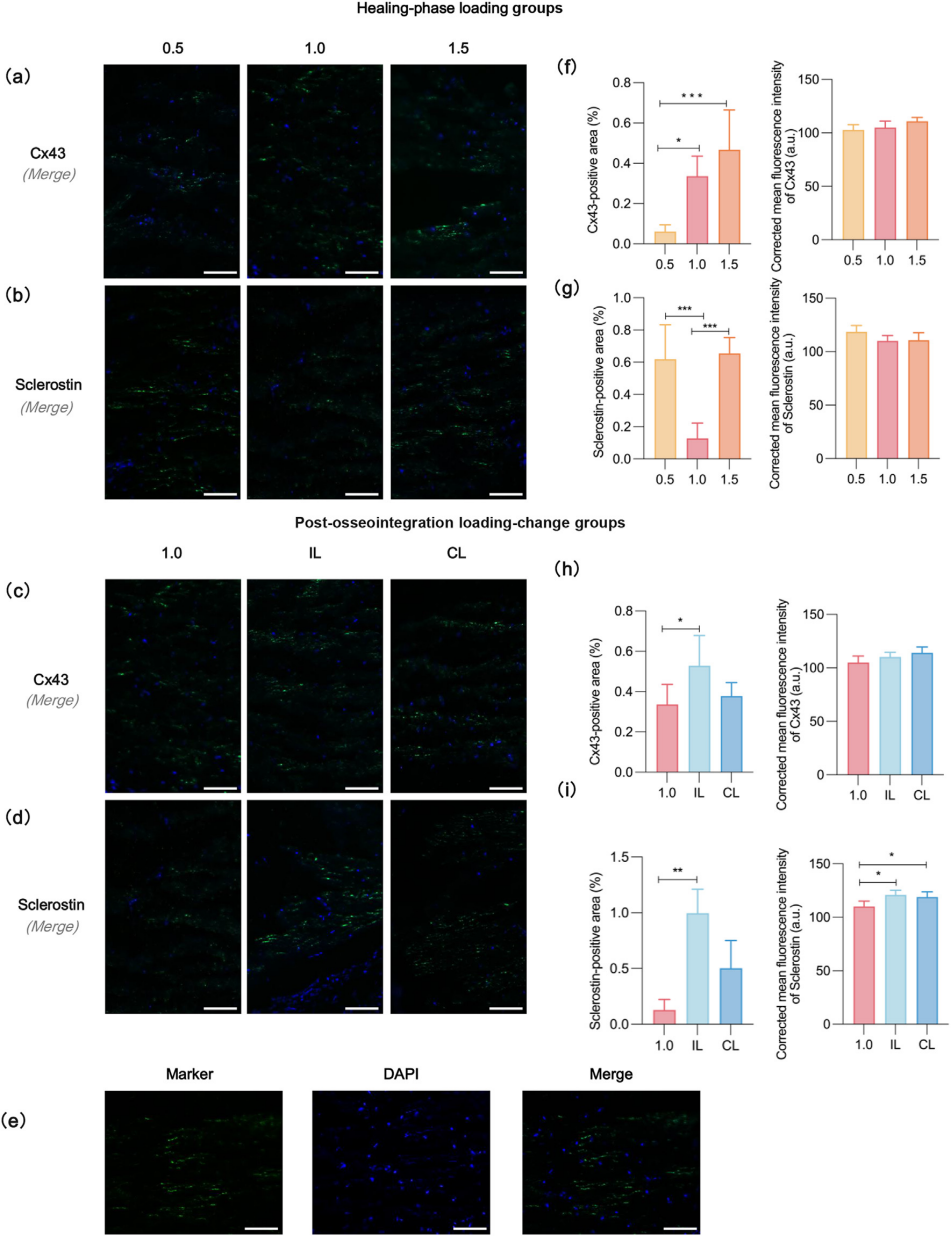
Immunofluorescence staining of Cx43 and sclerostin in peri**-**implant bone. Representative immunofluorescence images of Cx43 and sclerostin are shown for the healing-phase loading model and the post-osseointegration loading-change model (a-d). Because the 1.0 group served as the reference group for both models, the same representative immunofluorescence images are shown for the 1.0 group and the 1.0 control group, with the latter presented for comparison with the IL and CL groups. Scale bars, 50 μm. Representative single-channel and merged images are shown in (e), including target marker, DAPI, and merged signals. Quantitative results are shown for Cx43-positive area and corrected mean fluorescence intensity in the healing-phase loading model (f), sclerostin-positive area and corrected mean fluorescence intensity in the healing-phase loading model (g), Cx43-positive area and corrected mean fluorescence intensity in the post-osseointegration loading-change model (h), and sclerostin-positive area and corrected mean fluorescence intensity in the post-osseointegration loading-change model (i). Positive area (%) and corrected mean fluorescence intensity for Cx43 and sclerostin were quantified using Fiji. Positive area was calculated as the percentage of positive signal area within the ROI. Corrected mean fluorescence intensity was calculated as mean fluorescence intensity minus background intensity. Data are presented as mean ± SD. n = 5 animals per group for each analytical dataset. *P < .05, **P < .01, ***P < .001. Cx43, connexin 43; IL, ipsilateral change in occlusal loading; CL, contralateral change in occlusal loading.

In the healing-phase loading groups, Cx43 immunofluorescence was primarily localised in osteocyte cell bodies and dendritic processes (Fig. 6a). Cx43-positive area differed significantly among the 0.5, 1.0 and 1.5 groups (0.5: 0.062 ± 0.032%; 1.0: 0.336 ± 0.099%; 1.5: 0.467 ± 0.198%; one-way ANOVA, *P* = .001; Fig. 6f), with higher values in the 1.0 and 1.5 groups than in the 0.5 group (Tukey, *P* = .015 and *P* = .001, respectively). Cx43 corrected mean fluorescence intensity did not differ significantly among the groups (*P* = .061). Sclerostin-positive area also differed significantly among the groups (0.5: 0.619 ± 0.213%; 1.0: 0.127 ± 0.096%; 1.5: 0.655 ± 0.098%; one-way ANOVA, *P* < .001; Fig. 6g), with a lower value in the 1.0 group than in both the 0.5 and 1.5 groups (Tukey, *P* = .001 and *P* < .001, respectively). Sclerostin corrected mean fluorescence intensity did not differ significantly among the groups (*P* = .087).

In the post-osseointegration loading-change groups, Cx43 immunofluorescence showed a relatively continuous intercellular distribution pattern (Fig. 6c). Cx43-positive area differed significantly among the 1.0, IL and CL groups (1.0: 0.336 ± 0.099%; IL: 0.529 ± 0.150%; CL: 0.378 ± 0.066%; one-way ANOVA, *P* = .042; Fig. 6h), with a higher value in the IL group than in the 1.0 group (Tukey, *P* = .043). Cx43 corrected mean fluorescence intensity did not differ significantly among the groups (*P* = .051). Sclerostin-positive area differed significantly among the groups (1.0: 0.127 ± 0.096%; IL: 0.996 ± 0.215%; CL: 0.502 ± 0.249%; Kruskal–Wallis test, *P* = .005; Fig. 6i), with a higher value in the IL group than in the 1.0 group (Dunn-Bonferroni, *P* = .003). Sclerostin corrected mean fluorescence intensity was significantly higher in both the IL and CL groups than in the 1.0 group (1.0: 110.13 ± 5.05; IL: 120.90 ± 4.35; CL: 118.97 ± 4.95; one-way ANOVA, *P* = .009; Tukey, *P* = .010 and *P* = .032, respectively).

## Discussion

This study demonstrated stage-dependent peri-implant bone responses to occlusal contact-related conditions within the same experimental framework. During healing, increased peri-implant bone formation was not necessarily accompanied by better interface continuity, as the 1.5 group showed higher BA% and ALP-positive area but reduced BIC%. After initial osseointegration, the IL and CL groups showed reduced BIC% and increased TRAP-positive area without a statistically detectable reduction in BA% over 1 week. These findings suggest that peri-implant bone responses to occlusal contact-related conditions depend on interface maturity and the timing of loading-related changes, supporting the concept that implant occlusion should be viewed as a dynamic functional environment throughout healing, restoration and maintenance.

The healing-phase findings help explain why early functional conditions should be controlled during bone–implant interface formation. The 0.5 group maintained relatively continuous bone–implant contact but showed lower peri-implant bone area, suggesting that structural adaptation may have been limited under the lower contact-level condition. In contrast, the 1.5 group showed higher BA% and the highest ALP-positive area, but also reduced BIC%, reduced continuity of the crestal bone–implant interface, and soft tissue ingrowth around the implant neck. The 1.0 group therefore showed a comparatively balanced pattern, with greater BA% than the 0.5 group and better preservation of BIC% than the 1.5 group. This interpretation was further supported by the exploratory immunofluorescence findings: Cx43-positive area was higher in the 1.0 and 1.5 groups than in the 0.5 group, suggesting broader involvement of osteocyte-related mechanosensitive regions, whereas sclerostin-positive area was lower in the 1.0 group than in both the 0.5 and 1.5 groups, a pattern compatible with a relatively more favourable osteocyte regulatory environment during interface formation. Overall, these findings do not define an optimal loading threshold, but suggest that the 1.0 contact-level condition provided a more favourable balance among peri-implant bone formation, osteocyte-related regulation and bone–implant interface continuity within this experimental model.

The findings in the 1.5 group indicate that increased peri-implant bone formation should not be interpreted as improved osseointegration. Although higher BA% and ALP-positive area may reflect reactive osteogenic adaptation to mechanical stimulation,^18^^,^^37^ osseointegration requires stable and continuous bone contact along the implant surface. Biomechanical factors such as crown-height space, lever arms and contact position may influence peri-implant load transfer.^4^^,^^9^^,^^10^ Therefore, the higher contact-level condition may have promoted bone formation while disturbing the immature interface, contributing to reduced BIC%.

The osteocyte-related immunofluorescence findings during healing should be interpreted in relation to the contact-level–dependent remodelling pattern. The distinction between positive area and corrected mean fluorescence intensity should also be considered: positive area reflects the spatial extent of marker-positive regions, whereas corrected mean fluorescence intensity reflects relative signal strength within the measured regions. During healing, Cx43-positive area increased in the 1.0 and 1.5 groups without a significant increase in corrected mean fluorescence intensity, suggesting broader spatial involvement of Cx43-positive osteocyte regions rather than local signal amplification. In parallel, sclerostin-positive area, rather than corrected mean fluorescence intensity, differed among the groups, with the lowest value in the 1.0 group. Because sclerostin is involved in mechanically mediated bone adaptation and mechanical loading is generally associated with Sost/sclerostin downregulation and Wnt-related osteogenic responses,^20^^,^^21^ the lower sclerostin-positive area in the 1.0 group may be compatible with a more favourable osteocyte regulatory environment during interface formation. Conversely, the broader sclerostin-positive distribution in the 1.5 group, together with increased ALP-positive area, higher BA%, reduced BIC%, and reduced continuity of the crestal bone–implant interface, may reflect osteocyte-mediated inhibitory or stress-related feedback under a less favourable contact-level condition. This interpretation is consistent with peri-implant evidence showing increased sclerostin expression under impact loading and microdamage.^22^ Together, these exploratory findings suggest contact-level-dependent osteocyte-related regulation, but they do not prove enhanced osseointegration potential or a complete mechanistic pathway.

After initial osseointegration, dentition alteration was accompanied by resorption-related remodelling at the bone–implant interface. The IL and CL groups showed reduced BIC% and increased TRAP-positive area, whereas BA% and ALP-positive area did not show statistically significant changes. This combination suggests an active interfacial remodelling state. Thus, loading-change conditions after osseointegration may first manifest as localised interfacial remodelling rather than as a detectable reduction in overall peri-implant bone area during the short observation period. This interpretation is consistent with animal evidence showing that peri-implant bone responses after osseointegration depend strongly on loading mode, magnitude, tissue condition and experimental protocol.^38^, ^39^, ^40^ Therefore, preserved peri-implant bone area cannot exclude biologically active changes at the bone–implant interface.

After osseointegration, the osteocyte-related immunofluorescence pattern also differed from that observed during healing. The Cx43 response was relatively limited: Cx43-positive area was higher in the IL group than in the 1.0 group, whereas corrected mean fluorescence intensity did not reach statistical significance. In contrast, sclerostin showed a more pronounced response: sclerostin-positive area was higher in the IL group than in the 1.0 group, and corrected mean fluorescence intensity was higher in both the IL and CL groups than in the 1.0 group. This sclerostin response, together with reduced BIC% and increased TRAP-positive area in the absence of a significant BA% reduction, supports the interpretation that preserved peri-implant bone area does not necessarily indicate preserved bone–implant interface continuity.

The similar responses observed in the IL and CL groups suggest that the implant loading environment may be influenced by more than local adjacent dentition. The CL findings further suggest that whole-arch occlusal support, masticatory patterns, and redistribution of functional loading may influence the peri-implant environment. Clinical studies have shown that occlusal contacts of posterior single-implant crowns may change over time and that occlusal changes may be associated with peri-implant bone levels; occlusal assessment has therefore been emphasised as part of implant maintenance.^6^, ^7^, ^8^ Together with the present IL and CL findings, these observations suggest that adjacent tooth loss, contralateral dentition alteration, changes in opposing dentition, prosthetic wear, screw loosening, ceramic chipping and parafunctional activity may alter load distribution and contribute to interfacial remodelling.^6^, ^7^, ^8^^,^^41^

The 2 experimental settings indicate different remodelling patterns according to interface maturity and the timing of loading-related changes. During healing, contact-level conditions were associated with formation-related adaptation, as reflected by increased BA% and ALP-positive area, whereas the higher contact-level condition was also accompanied by reduced BIC% and impaired interface continuity. After initial osseointegration, dentition alteration was characterised mainly by resorption-related interfacial remodelling, with increased TRAP-positive area and reduced BIC% but no statistically detectable reduction in BA% over the 1-week observation period. These differences may be related to interface maturity and exposure duration: the immature bone–implant interface during healing may be more vulnerable to mechanical disturbance, whereas dentition alteration after initial osseointegration may first manifest as localised resorptive remodelling. These findings should therefore be interpreted as stage- and time-dependent remodelling responses rather than contradictory findings.

This stage-dependent remodelling phenotype may also explain the apparently conflicting findings in the occlusal overload literature. Previous animal studies have reported divergent findings, ranging from no clear loss of osseointegration under healthy peri-implant tissue conditions to crater-like bone defects or responses modified by inflammatory status and loading protocol.^16^^,^^25^^,^^38^, ^39^, ^40^ These findings indicate that occlusal loading should not be interpreted simply as harmful or harmless. Its biological effect may depend on interface maturity, loading type, exposure duration, inflammatory status and the outcome used to define success or failure.

The clinical value of this study lies in shifting implant occlusion from a static adjustment concept to a dynamic maintenance concept. During healing, contact control should aim to protect the developing bone–implant interface rather than to maximise early functional loading. After osseointegration, dentition alteration may reactivate interfacial remodelling even when the overall peri-implant bone area appears preserved. For immediate or early restoration protocols, premature heavy contact, eccentric interference, impact loading and excessive crown-height space should be carefully controlled or minimised.^4^^,^^5^^,^^9^^,^^10^ During maintenance, follow-up should include reassessment of occlusal contact distribution, crown-height space, changes in adjacent, opposing and contralateral dentition, prosthetic wear, screw loosening, ceramic cracking or chipping, bruxism and other parafunctional risks.^8^^,^^41^ Together, these findings support stage-specific occlusal monitoring throughout healing, restoration and long-term function. However, because these findings were obtained from a preclinical rat model, they should be interpreted as biological evidence supporting stage-specific occlusal monitoring rather than as direct clinical loading recommendations.

The present findings should be interpreted within the limitations of a preclinical animal study. First, 5-week-old male Wistar rats differ from adult human patients in terms of bone turnover, masticatory patterns, jaw growth and sex-related biological responses. Second, the actual occlusal force magnitude, direction, frequency and duration were not measured directly; therefore, the healing-phase loading groups should be interpreted as contact-level conditions rather than as quantified loading groups. Third, the post-osseointegration loading-change groups lacked a surgery-matched sham control and a 4-week baseline sacrifice group, so the effects of secondary anaesthesia, extraction wound healing, altered feeding behaviour and load redistribution could not be fully separated. Fourth, histomorphometric, histochemical and immunofluorescence analyses were performed using different specimens, and two-dimensional section-based analysis cannot fully represent the three-dimensional structure of peri-implant bone. Fifth, the Cx43 and sclerostin findings should be interpreted as exploratory because independent positive-control tissue staining was not performed. Finally, removal torque testing, resonance frequency analysis, dynamic fluorochrome labelling, micro-CT and direct microdamage assessment were not performed. Therefore, these findings should be interpreted as mechanically related peri-implant bone remodelling responses in an in vivo oral environment rather than as evidence that occlusal loading alone causes peri-implantitis.

## Conclusion

Within the limitations of this rat intraoral model, bone responses around implants varied by treatment stage. Greater or preserved peri-implant bone area did not necessarily reflect continuous bone–implant contact, supporting the concept of bone area–interface decoupling. During healing, contact conditions were associated with bone formation but also interface vulnerability, whereas after osseointegration, changes in loading were associated with interfacial remodelling despite preserved bone area. These findings support stage-specific occlusal monitoring, with protection of the developing interface during healing and reassessment of occlusal risks during maintenance.

## Data availability

The data supporting the findings of this study are available from the corresponding author upon reasonable request.

## Author contributions

*Concept and design:* Ruolan Cao, Yoko Takemura. *Acquisition, analysis, or interpretation of data:* Ruolan Cao, Yoko Takemura. *Drafting of the manuscript:* Ruolan Cao, Yoko Takemura. *Critical revision of the manuscript for important intellectual content:* Yasuko Moriyama, Yasunori Ayukawa. *Administrative, technical, or material support:* Wenxuan Lai, Xi Chen, Ami Izumi, Mari Abe. *Supervision:* Yasuko Moriyama, Yasunori Ayukawa.

## Conflict of interest

None disclosed.
